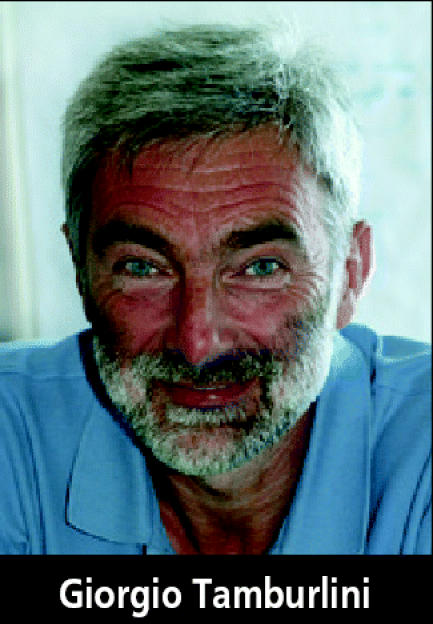# Editorial: Children’s Health and the Environment: A Transatlantic Dialogue

**DOI:** 10.1289/ehp.113-1281297

**Published:** 2005-10

**Authors:** Philip J. Landrigan, Giorgio Tamburlini

**Affiliations:** Department of Community & Preventive Medicine, Mount Sinai School of Medicine, New York, New York, E-mail: phil.landrigan@mssm.edu; Institute of Child Health, IRCCS Burlo Garofolo, Trieste, Italy

Important steps to protect children against environmental threats to health have been taken over the past decade in both the United States and Europe This progress is based on the shared recognition that infants and children are very different from adults in their exposures and their susceptibility to toxic chemicals and that they therefore require special protections in risk assessment, regulation, and law. But despite this common scientific foundation, developments in children’s environmental health (CEH) on the two sides of the Atlantic have been quite different. These contrasting and sometimes complementary advances reflect the differing social, legal, and regulatory cultures of the two continents.

Recognition among policy makers of the unique vulnerability of children had its origins in the United States and dates from the publication in 1993 of the National Research Council (NRC) report *Pesticides in the Diets of Infants and Children* (National Research Council 1993). This report found striking differences between children and adults in exposure as well as in susceptibility to toxic chemicals. The report identified large gaps in regulatory practice and called for expansion of toxicologic testing to assess threats to development. It also urged reform of risk assessment and regulation to enhance protection of children. The central contribution of the NRC report was to elevate consideration of the vulnerability of children from the specialized area of pediatrics to the broad realm of national policy formulation.

The recommendations of the NRC report were incorporated into federal policy in the United States in 1996 through the Food Quality Protection Act (FQPA), the principal U.S. statute governing use of pesticides (FQPA 1996). The FQPA affirms the unique vulnerability of children. It requires explicit consideration of children in risk assessment and mandates child-protective safety factors in regulation. These principles were reaffirmed in April 1997 in an Executive Order on Children’s Environmental Health and Safety requiring all agencies of the U.S. government to consider children’s health and safety in all policy decisions (Clinton 1997). This U.S. experience was shared internationally and led to promulgation in May 1997 of the Miami Declaration on Children’s Environment Health, a declaration approved unanimously by the environmental ministers of the G-8 nations (Environment Leaders' Summit of the Eight 1997).

In recent years, CEH policy development has slowed in the United States, but the nation continues to make important contributions in research and medical practice. These advances date from 1998, when the National Institute of Environmental Health Sciences and the U.S. Environmental Protection Agency established a national network of Centers for Children's Environmental Health and Disease Prevention Research, now 11 in number. These interdisciplinary centers have matured into strong generators of scientific knowledge, they have produced unprecedented gains in environmental pediatrics, and they have been effective incubators of the careers of young scientists and physicians who will become the next generation of leaders in CEH.

Research in the centers has made important contributions to understanding of the environmental causes of asthma, neurobehavioral disorders, endocrine dysfunction, autism and low-level lead toxicity. Findings from this work are already guiding disease prevention. Reports highlighting the accomplishments of the centers are presented in this issue of *Environmental Health Perspectives*.

The National Children’s Study (NCS) is a second emerging research development in the United States (National Children’s Study 2005). The NCS is a prospective epidemiologic study that will follow 100,000 children—a statistically representative sample of all children born in the United States—from (or before) conception to 21 years of age. The goal is to identify the factors in the environment—chemical, biologic, physical, and psychosocial—that alone or in combination influence children’s health, growth, development, and risk of disease (Trasande and Landrigan 2004).

In pediatric practice, a major U.S. innovation has been development of a network of Pediatric Environmental Health Specialty Units (PEHSUs) [Agency for Toxic Substances and Disease Registry (ATSDR) 2005]. This network now includes 11 sites across the United States, and PEHSUs have also been established in Canada, Mexico, Spain, and (soon) Argentina. PEHSUs are clinical units designed to diagnose and treat children with diseases of toxic environmental origin, to improve access to expertise in pediatric environmental medicine, and to educate health care practitioners about environmental threats to children’s health.

In Europe, progress in protecting children against environmental threats initially lagged behind that in the United States. In recent years, however, advances in Europe have leapfrogged ahead.

CEH first emerged as a policy issue in Europe in 1999 at the third Ministerial Conference on Environment and Health, held in London. This Conference cited the Miami Declaration (Environment Leaders' Summit of the Eight 1997) and emphasized the importance of protecting children from environmental exposures. It identified priority areas for action and started a process that lead to the Fourth Ministerial Conference.

The Fourth Ministerial Conference, held in Budapest in 2004, was a watershed event for CEH. Preparation was coordinated by the World Health Organization (WHO) European Office and the European Environment and Health Committee. It involved eight large meetings with active involvement of all 52 member states of the WHO European Region, as well as nongovernmental organizations and representatives of industry and trade unions. The goal was to define the rationale, structure, and objectives of a novel Children’s Environment and Health Action Plan for Europe (CEHAPE; Ministers of Health and Environment in the European Region of the WHO 2004). Two major products of this process were *a*) a thorough review of the scientific evidence on children’s environmental health, published by the WHO European Office and the European Environment Agency (Tamburlini et al. 2002); and *b*) a study that quantified for the first time the burden of disease in children and adolescents in Europe related to environmental exposures (Valent et al. 2004).

At the Budapest Conference, CEHAPE was approved at the highest political level (Valent et al. 2004), thus affirming the commitments of all 52 European member states to mitigation of environmental threats to children’s health. An important feature of CEHAPE is its recognition that children in particularly adverse conditions, such as war or extreme poverty, are at highest risk of injuries, psychological trauma, acute and chronic infections, chronic diseases, disability, and death. CEHAPE urges that special emphasis be placed on preventing these conditions and their risk factors.

The challenge now confronting the European member states is to implement CEHAPE. This work is being coordinated by the WHO European Office–CEH Unit in Rome. Policy tools guiding this process include a book summarizing evidence on children’s environmental health; tools showing countries how to transform the CEHAPE framework into national action plans; a compilation of successful experiences in prevention; and a set of CEH indicators (Licari et al. 2005; Nemer and von Hoff, in press).

Following the commitments made in London and Budapest, the European Commission (EC) has strengthened its focus on CEH. It has provided funding to Member States for implementation of CEHAPE, and has developed far-reaching policies and action plans:

- REACH (Registration, Evaluation and Authorization of CHemicals)
- SCALE (Science, Children, Awareness raising, Legislation, Evaluation)
- The European Environment and Health Strategy
- The 2004–2010 Environment and Health Action Plan.

REACH, proposed by the EC in October 2003, is the most ambitious of these proposals (European Commission 2003). It presents a new European regulatory framework for chemicals, and its goal is to close the current gap in knowledge of the toxicity of chemicals. REACH requires that safety and toxicity information be made publicly available on all chemicals produced or imported in Europe in volumes > 1 ton/year per manufacturer/importer. Under REACH, the burden of proof to establish the safety of a chemical will be on industry. Innovation of safer substances will be encouraged under REACH by providing exemptions for research and development. If fully adopted, REACH will hasten the end of the vast ongoing toxicologic experiment in which chemicals are being tested on children worldwide instead of in the laboratory.

REACH is currently under examination by the European Parliament. In a recent public hearing, the current EC—which seems more attentive than its predecessor to the concerns of industry—discussed the importance of balancing children’s health against the competitiveness of European industry.

Among researchers and pediatric practitioners in Europe, CEH seems to be gaining momentum, though at a slower pace than in the United States. CEH is increasingly a focus of epidemiologic investigation, with recent studies examining the effects on children of air pollution and neurotoxicants. Large prospective cohort studies of children are under way in several nations (for example, in the United Kingdom and the Nordic countries) or are being started (Italy). A new research consortium focusing on CEH has been established in Trieste, Italy, under the leadership of the Children’s Hospital.

The evolution of research, practice, and policy in CEH on the two sides of the Atlantic has been a fascinating and interconnected process. Great progress has been made, but this progress raises questions. What, for example, will be the impact of European policy initiatives on policy in the United States? Will adoption of REACH puncture U.S. complacency on chemical testing? Will CEHAPE prod the United States into setting benchmarks and national goals for CEH? And what will be the impacts on policy in Europe of the new science now emerging from the Children’s Environmental Health Centers in the United States and soon to come from the National Children’s Study? Will the information emerging from this research result in bans or restrictions on classes of chemicals?

And what of the Developing World? Will the new science and policies emerging from the industrially developed nations influence the industrializing countries? In this time of globalization, the nations of the Developing World host ever more hazardous industries and ever more toxic chemicals, as those industries and chemicals become more and more unwelcome on the two sides of the North Atlantic. What will be the consequences?

These are critically important questions. If we consider protection of the health of our children an important value, we must confront them.

## Figures and Tables

**Figure:**
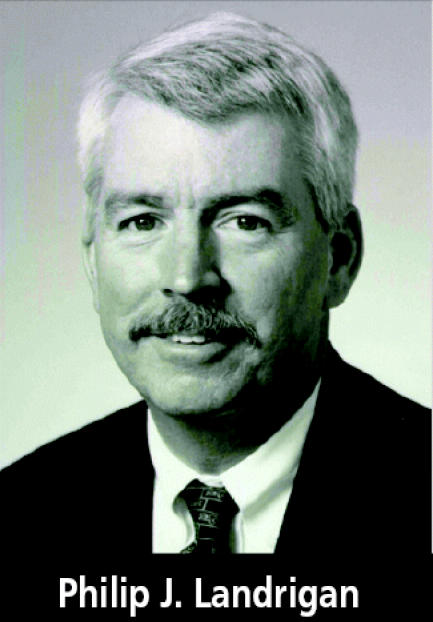


**Figure:**